# Complete Genome Sequence of *Bacteroides ovatus* V975

**DOI:** 10.1128/genomeA.01335-16

**Published:** 2016-12-01

**Authors:** Udo Wegmann, Alexander Goesmann, Simon R. Carding

**Affiliations:** a Gut Health and Food Safety Programme, Institute of Food Research, Norwich Research Park, Norwich, United Kingdom; b Norwich Medical School, University of East Anglia, Norwich Research Park, Norwich, United Kingdom; c Bioinformatics and Systems Biology, Justus-Liebig-University, Giessen, Germany

## Abstract

The complete genome sequence of *Bacteroides ovatus* V975 was determined. The genome consists of a single circular chromosome of 6,475,296 bp containing five rRNA operons, 68 tRNA genes, and 4,959 coding genes.

## GENOME ANNOUNCEMENT

The human gastrointestinal tract hosts a plethora of resident microorganisms with bacterial cell densities in the colon reaching 10^11^ cells per g of content (1). Although an individual’s microbiota is unique and variable, a dominant phylogenetic core (2) consisting of members of the phyla *Firmicutes* and *Bacteroidetes* constitutes up to 90% of the colonic microbiota in all human populations (3, 4). Among the *Bacteroidetes*, representatives of the genus *Bacteroides* are among the most abundant bacterial species of the human colonic microbiota, of which *Bacteroides ovatus*, a Gram-negative, rod-shaped, non-spore-forming, and anaerobic bacterium, exists in more than 90% of individuals (5). As a member of the *Bacteroidetes* phylum, *Bacteroides* spp. diverged from the common line of eubacterial descent before the major eubacterial groups and are distinct from the other major Gram-negative phylum, the *Proteobacteria* (6). Their membranes contain sphingolipids (7), and the structure of their promoters (8) and ribosomal binding sites (9) is distinct from that of proteobacteria. Recent advances in the generation of genetic tools for *Bacteroides*, their prevalence among human populations, and the fact that these organisms are among the most stable components of the human gut microbiota (10) have led to the proposed use of genetically modified *B. ovatus* as a vehicle for the delivery of therapeutic agents in humans (11). This requires the complete genome sequence of the bacterium to facilitate its genetic manipulation and second, to evaluate its suitability with regard to biological and clinical safety. Hence, we undertook sequencing of the genome of *B. ovatus* V975.

The complete genome sequence was determined using the Genome Sequencer FLX 454 system. The initial draft assembly provided by MWG-Biotech (Ebersberg, Germany) was based on a total of 488,596 pyrosequencing reads, with an average read length of 278 nucleotides (nt), and included 146,170 reads which had been generated following the long paired-end tag protocol. After Newbler assembly and contig ordering based on paired-end reads, the 6,489,366-Mbp draft assembly consisted of 44 contigs, which were distributed across 33 scaffolds, and the average per-base coverage was 23-fold. Standard PCR, followed by primer walk sequencing on the resulting products, was used to close the gaps located in scaffolds. Multiplex PCR was employed to identify adjoining contigs and respective primer pairs for which no linkage had been established previously, and upon reamplification under standard conditions, the resulting products were analyzed by primer walk sequencing. The sequence assembly was carried out with the Staden package (12), and the integrity of the assembly was confirmed by pulsed-field gel electrophoresis of restricted agarose embedded DNA in a CHEF-DR II electrophoresis system (Bio-Rad Laboratories, Hercules, CA), according to the manufacturer’s instructions. The finished *B. ovatus* sequence was annotated using the GenDB 2.4 annotation tool (13).

The genome consists of a single circular chromosome of 6,475,296 bp, with an average G+C content of 41.88%. It contains five rRNA operons, 68 tRNA genes, and 4,959 coding genes.

### Accession number(s).

The genome sequence has been deposited at the European Nucleotide Archive under the accession number LT622246.
